# The perspectives of healthcare practitioners on fall risk factors in older adults

**DOI:** 10.4102/hsag.v25i0.1495

**Published:** 2020-12-04

**Authors:** Hendrika de Clercq, Alida Naude, Juan Bornman

**Affiliations:** 1 Centre for Augmentative and Alternative Communication, Faculty of Humanities, University of Pretoria, Pretoria, South Africa

**Keywords:** disability and health, falls, fall risk, focus groups, ICF, International Classification of Functioning, healthcare practitioners

## Abstract

**Background:**

Accidental falls could have severe and far-reaching consequences for older adults, their families and society at large. Healthcare practitioners’ (HCPs) perspectives on fall risk factors in older adults could assist in reducing and even preventing falls. Currently, no universal tool exists for this purpose. The World Health Organization’s globally accepted International Classification of Functioning, Disability and Health (ICF) was used.

**Aim:**

This study aimed to (1) describe the perspectives of HCPs on fall risk factors in older adults in South Africa and (2) link these factors to the ICF.

**Setting:**

Eighteen HCPs participated in two focus groups.

**Methods:**

Using a qualitative research design, an inductive thematic analysis allowed for the identification of important themes, which were linked to the ICF.

**Results:**

The factors mentioned by participants were categorised into 38 themes, which were linked to 142 ICF codes, of which 43% (*n* = 61) were linked to the Body Function category, 23% (*n* = 32) to the Environmental Factors category, 18% (*n* = 26) to the Body Structure category and 16% (*n* = 23) to the Activities and Participation category. HCPs revealed two relevant factors that were not captured in existing fall risk assessment tools (FRATs), namely ‘muscle-power functions’ and ‘mobility-of-joint functions’, which directly relate to the ability to execute mobility activities. Combining HCPs’ perspectives with other stakeholders and with literature provides a holistic picture of fall risk factors in older adults.

## Introduction

Accidental falls are the leading cause of injury-related deaths amongst older adults (National Center for Injury Prevention and Control 2019; i.e. individuals older than 65 years of age). Falls are therefore considered one of the five so-called ‘geriatric giants’, along with dementia, poor mobility, incontinence and polypharmacy (Cumming 2013). Internationally, it is estimated that a third of community-dwelling older adults may experience accidental falls every year, with a potential 35.5% experiencing recurrent falls (Hung et al. 2017). Although research on falls in South Africa is scarce (Kalula et al. 2016), a recent study revealed that falls are the most common injury in older adults for which medical intervention is required (Da Costa 2020). On the African continent, South Africa has one of the highest proportions of older adults, compared with other African countries, such as Angola, Burkina Faso, Gambia and Uganda. This population is expected to grow from 4 million in 2011 to over 10 million in 2030 (Kelly, Mrengqwa & Geffen 2019). Falls in older adults could have severe and far-reaching consequences, not only for the person who falls but also for their family members – as a third-party disability (Hickson & Scarinci 2007) – and for society at large. The International Classification of Functioning, Disability and Health (ICF) describes third-party disability as the disability experienced by significant others as a consequence of their family members’ health condition (in this case falls), and the impact this has on the family members’ life functioning. As such, falls have several financial and environmental consequences, for instance hospitalisation and early nursing home admission, which may result in a socio-economic burden on the person who falls, family members and the healthcare system (Bird et al. 2013; Howcroft, Kofman & Lemaire 2013; Liu et al. 2017).

The majority of the South African population relies on the public healthcare system – public hospitals, clinics and medical facilities – for medical intervention. The public health system in South Africa is an institution under severe restraint, with a shortage of healthcare practitioners (HCPs), poor leadership and often improper allocation and use of the limited resources (Kelly et al. 2019). Healthcare access, in both the public and the private sectors, is shaped by several factors, including the characteristics, financial position, social capital and level of education of the population and the limited availability of equipment, medicine, skilled HCPs and facilities (Kelly et al. 2019). Although the private healthcare system in South Africa is currently better equipped than the public system with both resources and HCPs, older adults nonetheless experience difficulties in navigating their medical aid funds to cover HCP fees. They also struggle to afford medical co-payments and cope with having to wait several weeks to months for specialist appointments. Because community-dwelling older adults face challenges in accessing appropriate care and support from medical facilities, they often under-utilise the healthcare system or receive insufficient care (Kelly et al. 2019). Because early identification of fall risk factors may potentially decrease the rate of falls in older adults, HCPs could assist in reducing older adults’ chances of landing in the healthcare system for falls and fall-related injuries. Healthcare practitioners’ play an important role in relieving not only the burden on the healthcare system but also, more importantly, the burden on older adults who have to live with the negative consequences of fall-related injuries. The latter could lead to reduced functioning and decrease these older persons’ health-related quality of life (HRQoL; Fahlström et al. 2018). By lowering their fear of falling and improving their physical functioning, HCPs could help to reduce fall risks even more, thus creating a positive and upward spiral of health in older adults (Bjerk et al. 2018).

Despite the relatively small percentage of older adults in South Africa (8.6%) – considering a population size of almost 60 million in 2020 – this translates into an actual figure of more than 5.2 million older adults (South African Government 2018). With the expected growth of this population, as well as the burden that falls in older adults could have on the healthcare system and society at large, it is important to determine the perceptions of HCPs on fall-related risk factors. It is also imperative to use this knowledge to potentially increase early identification of fall risk factors in older adults and so reduce falls and fall-related injuries in this population. Literature is abundant with different factors that could potentially increase this population’s risk of falling, although most studies are aimed at hospital settings or at specific medical conditions, e.g. Parkinson’s disease or cancer (Myers 2013; Park 2017; Voss et al. 2012). A recent study (Howland et al. 2018) indicated that although almost all HCPs in their sample (*n* = 97) believed that older adults should regularly be screened (as this could guide HCPs to implement prevention strategies to reduce fall risk in older adults), only half of them felt confident to perform fall risk screenings in this population. Furthermore, these HCPs did not believe that conducting fall risk screenings currently constitutes the prevailing standard of practice in their profession. Some of the reasons for not screening for fall risk as routine practice could relate to limited time to complete such screenings during routine consultations (Hunderfund et al. 2011), not necessarily being compensated for these screening procedures by the healthcare system or medical aids (Howland et al. 2018) and the sheer amount of available screening fall risk assessment tools (FRATs). Another reason may be the fact that FRATs are currently not well-integrated as routine clinical practice in the majority of HCP practices (Howland et al. 2018). Identifying from a clinical point of view, the factors that are most relevant to fall risk in older adults could help to further develop early identification and screening methods to reduce falls in this population.

Reducing and even preventing fall risk in older adults firstly hinges on effective documentation of risk factors, which could then be used to guide further intervention and mitigate fall risk (Reinoso, Mccaffrey & Taylor 2018). One of the key strategies for compiling a list of relevant ICF codes involves determining the perspectives of HCPs on fall risk factors in older adults. Practitioners have an important role to play in identifying fall risk (Liddle et al. 2018), yet their perspectives are not routinely included in research on the topic (Burgon et al. 2019). Currently, no universal tool exists for HCPs to comprehensively document fall risk factors in older adults, and this possibly contributes to the ineffective management of the problem (De Clercq, Naude & Bornman 2020). One strategy towards creating an early identification documentation system is to develop a holistic, universal classification of fall risk in older adults. This can be done by incorporating the ICF as a framework for determining functioning in older adults who have a risk of falling and using this framework to guide the early identification of fall risk by HCPs of these patients. The World Health Organization’s ICF uses a universal language to ensure that the functioning of a person of any age, participating in any activity, can be documented in any healthcare setting by HCPs from different professional disciplines. Built on such a multidimensional view of functioning, the ICF is especially suitable to obtain health information because it recognises the individual (consisting of a body participating in specific activities) as being influenced by different contextual factors. As such, the ICF consists of three domains, namely Body Functions and Structures, Activities and Participation and Contextual Factors (which are divided into Environmental and Personal Factors). Several codes describe each domain, save for Personal Factors, which are not coded in the ICF. The complete ICF consists of more than 1400 codes, presenting a challenge in using it in clinical practice (Aiachini et al. 2010). Therefore, researchers and clinicians alike have recommended that the most relevant and typical codes should be determined for a specific condition – termed a ‘code set’ – as that would enable HCPs to utilise the ICF more effectively in clinical practice. Currently, a code set does not exist to describe and document fall risk in older adults.

Based on their clinical experience, HCPs could offer new insights into the current knowledge on fall risk factors in older adults, thereby identifying potential risk factors not previously documented. Healthcare practitioners are also able to influence patients’ opinions on falls and to reduce fall risk (Burgon et al. 2019). Obtaining qualitative data on their perspectives on fall risk factors in older adults and linking these factors to the ICF as a universal framework could give insight into the clinical manifestation of fall risk in this population. The data could also be used to move towards incorporating the perspectives of HCPs into future fall risk guidelines for clinical practice. This could also help to identify areas to be considered in the compilation of a list of ICF codes, as well as to develop improved strategies to manage (Loganathan et al. 2015) and ultimately have a positive impact on older adults’ HRQoL. Healthcare practitioners are key stakeholders in the process of translating findings from literature and research into clinical practice and policies (Van Rhyn & Barwick 2019). By gathering their insights, researchers could develop more user-friendly and appropriate clinical tools for HCPs for use in their routine screening of these patients.

This study aims to fulfil two distinct objectives: firstly, to provide insight into the perspectives of HCPs in the South African context regarding risk factors associated with falls in older adults, and secondly, to link these factors to the ICF as a universal framework for describing functioning. The researchers hoped to move towards incorporating the perspectives of HCPs as key stakeholders into future fall risk guidelines for clinical practice.

## Method

Following a qualitative design, two focus group discussions were conducted, as these allowed the gathering of in-depth, detailed information on a novel topic – the perspectives of HCPs in South Africa on fall risk factors in older adults. This method ensured that all voices in the discussion were heard, thereby enhancing contemporary knowledge (Carey & Asbury 2012).

### Participants

#### Recruitment

As the *Protection of Personal Information (POPI) Act* prohibits the Health Professions Council of South Africa (HPCSA) to provide the contact details of currently practising HCPs to researchers, an internet search was conducted to identify potential facilities with multidisciplinary teams from both the public and the private sector, by using a convenience sampling method. Search terms included ‘frail care facilities Gauteng’, ‘multidisciplinary facilities Gauteng’, ‘holistic healthcare facility Pretoria’ and ‘public hospitals Gauteng’. Ten facilities – six private and four public facilities – were identified in the same geographical area and subsequently contacted telephonically. The research study was explained to the relevant authority figures, and they were invited to have the HCPs in their facility to participate. Of the 10 facilities, 5 agreed to consider the proposal, and eventually 2 of the relevant authority figures consented to their facility’s participation. Twenty-five potential participants were identified and a total of 18 participants consented; 8 of these participants were practising in the public sector and 10 in the private sector. The two venues that were chosen were easily accessible to the majority of participants in each sector. Two focus group discussions were held, one at a local public hospital, in the boardroom where weekly meetings are held, and one at a private institution, where approximately half of the participants worked. The ideal size of focus groups is described as being between 5 and 10 participants per group (Jacobsen 2021), and in this study, the first focus group had 10 participants and the second group had 8 participants.

#### Participant selection

Participants were selected based on their registration with either the HPCSA or the South African Nursing Council (SANC). They had to have at least 3 years of experience in their profession and at least 2 years of experience working with older adults. Experienced HCPs were more likely to be confident in their own knowledge and abilities, and hence they could be expected to contribute meaningfully to the discussions (Femdal & Solbjør 2018).

Because no consensus existed regarding the disciplines that would typically constitute a fall risk management team, an internet search for international fall clinics/centres was conducted to determine the most prominent disciplines involved. Based on the clinics’ websites and publicly available information, the following six disciplines were included in this study:

1. Medical practitioners (they educate patients regarding health and personal factors that cause falls) (Phelan et al. 2015).
2. Nurses (they typically screen and then refer patients for a more in-depth assessment) (Unsworth 2003)
3. Podiatrists (they focus on foot health care, patient education, health promotion, rehabilitation and mobility) (Frankowski 2010).
4. Physiotherapists (they can assess environmental and behavioural factors that cause falls or increase fall risk) (Sherrington & Tiedemann 2015).
5. Occupational therapists (they review patients’ home and work environments for hazards and evaluate their personal and environmental limitations that contribute to falls) (American Occupational Therapy Association 2020).
6. Audiologists (they identify, diagnose and provide treatment options for patients with vestibular disorders that lead to dizziness and imbalance, including fall risk) (Republic of South Africa 2009).

#### Participant description

All 18 participating HCPs were part of established multidisciplinary teams. They included two ear, nose and throat (ENT) specialists, two general practitioners (GPs), three nurses, three podiatrists, three physiotherapists, three occupational therapists and two audiologists. On average, the participants had 16 years’ experience in their current profession (ranging from 3 to 40 years), with an average of 14 years’ experience working with older adults (ranging from 2 to 39 years). The majority were female (*n* = 14).

**FIGURE 1:**
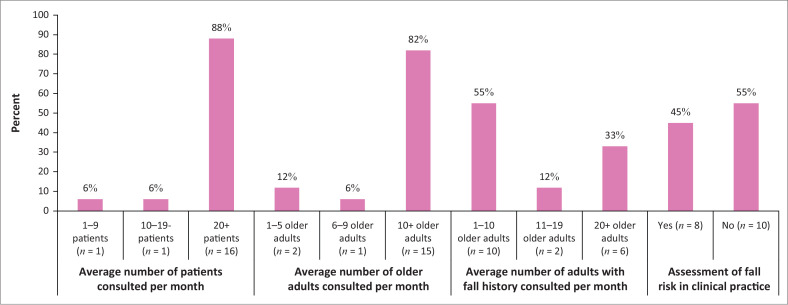
Service delivery by participants (average per month).

Figure 1 shows that 88% (*n* = 16) of the participants consulted at least 20 patients per month in their practice, 82% (*n* = 15) consulted at least 10 older adults per month in their practice and 55% (*n* = 10) of the participants consulted up to 10 older adults with a fall history per month. Just over half of the participants (55%; *n* = 10) indicated that they assess fall risk in the patients with whom they consult in their practices. However, during the discussions, all of the participants agreed that they assess fall risk in an informal manner only, or by asking the patient to perform certain tasks (e.g. standing in tandem or walking down the corridor). The occupational therapists and the nurses in the public hospital indicated that they use some of the elements of two popular FRATs (Berg Balance Scale and Morse Fall Scale) as part of an informal assessment of patients with a potential fall risk.

### Materials and equipment

Biographical questionnaires were compiled based on the inclusion criteria and were completed prior to the focus group meetings to ensure that all potential participants met the selection criteria as well as to obtain descriptive information (Sargeant 2012). A focus group script was followed to structure the group and ensure that the discussion would remain focussed and consistent across the two groups, thereby heightening procedural consistency and data integrity (Hennink 2014). The script contained specific steps for conducting the focus groups, as well as a specific question, namely ‘Which factors do you think can increase or decrease an older adult’s chance of falling?’ The open-ended question was broad enough to ensure a wide variety of answers. After discussing the main question, the participants were asked to consider fall risk factors that they thought HCPs could assess in clinical practice. Healthcare practitioners normally use critical thinking skills when they reflect on knowledge derived from interdisciplinary subject areas (Zayapragassarazan et al. 2016). Thus, they are able to relate the topic at hand to their own knowledge and experience in the assessment and intervention of patients they see on a regular basis (The Health Foundation 2012). By asking the participants to relate fall risk factors to their own experience in the assessment of their patients, the researcher was able to prompt more critical consideration of the relevant factors and enrich the data gathered during the focus group discussions. The materials and equipment enabled the researcher to gain insight into the perspectives of HCPs regarding fall risk factors in older adults.

### Data collection procedures

Prior to data collection, the relevant ethics permission was obtained from the University of Pretoria. All participants completed the informed consent forms and biographical questionnaires before commencement of the focus group. The aim of the research was explained in the focus group script, all questions that arose were discussed, and participants were alerted to the fact that the discussion would be audio-recorded and notes be made during the discussion. Everyone introduced themselves, and as most of the participants knew each other, rapport was quickly established.

Member checking was performed at the end of the focus groups by reading a summary of the main discussion points back to the participants and providing them the opportunity to clarify, alter or add to their contributions. Minimal clarifications were needed in both focus groups. On completion of the second focus group meeting, data saturation was reached. No new data were gathered compared with the first discussion, and there was no notable difference between the two groups that could have influenced the data (Fusch & Ness 2019). Data obtained from the two groups were thus collapsed into a single data set.

### Rigour

Participants were recruited from the same disciplines, but from different employment contexts, to ensure that multiple perspectives were obtained. Participant verification in the form of member checking was carried out, which is considered a crucial technique for establishing credibility in qualitative studies (Birt et al. 2016). It also facilitates a shared understanding and improves the accuracy of the data collected (Harper & Cole 2012). Data were analysed by using ATLAS.ti software, which enabled the complex organisation and retrieval of data and improved the rigour of analysis (Pope, Ziebland & Mays 2000). All three authors independently reviewed the themes as well as the codes linked to the ICF. After discussion, they fully agreed on the themes and the ICF codes to which each theme had been linked, thus resulting in a 100% inter-coder agreement score.

### Data analysis procedures

Two consecutive data analysis procedures were employed during this study. Firstly, an inductive thematic analysis was conducted to address the first objective as suggested by Clarke and Braun (2017). The five steps of data categorisation included (1) familiarisation with the data by reading and rereading the verbatim transcriptions; (2) assigning preliminary codes; (3) searching for themes by the researcher (HdC); (4) reviewing themes by all three authors; and (5) defining and grouping similar themes together. The three authors eventually agreed on the final list of themes.

Secondly, to address the second objective, the identified themes were linked to the ICF by means of a deductive data analysis in the form of a directed content analysis, by using the ICF linking rules (Cieza 2019). For the purposes of this study, a two-level ICF classification was sufficient and its first seven linking rules were utilised:

1. Acquiring good knowledge of the conceptual fundamentals of the ICF – that is by studying the ICF manuals and coding system prior to data analysis.
2. Identifying the main concept of each of the themes that would be linked to the ICF – that is in ‘walking outside on the sidewalks’, the main concept would be ‘walking’.
3. Identifying any additional concepts for each theme that could also be important and should be considered when linking the theme to the ICF – that is additional concepts to the previous example would be ‘outside’ and ‘sidewalks’.
4. Considering the popular perspectives for each identified concept and whether the perspectives on the theme influenced the intended meaning of the theme – that is by reading current literature on the topic at hand.
5. Identifying and documenting all the identified, meaningful concepts that would be linked to the ICF – i.e. all main and additional concepts were listed with the number of times each concept was mentioned.
6. Linking all the meaningful concepts to the precise ICF category – i.e. ‘walking’ would be linked to the ICF code ‘moving around in different locations’.
7. Using ‘other specific’ or ‘unspecified’ ICF categories as appropriate.

All the meaningful concepts and linked codes of the identified themes were independently reviewed by the three authors, and an initial inter-coder agreement score of 98% was established. After discussion, full agreement on all the linked codes was established.

### Ethical consideration

The study was approved by the Research Ethics Committee of the University of Pretoria, South Africa (Ethics Approval Reference Number: GW20170917HS).This study followed the ethical considerations as set out by Declaration of Helsinki (World Medical Association 2001), including the principles of informed consent, voluntary participation, deception and clinical use, confidentiality and respect, social use and objectivity and professional integrity.

## Results

The focus group participants provided rich insights into their perspectives with regard to the fall risk factors that they considered relevant in older adults. Table 1 lists the points that emerged from these discussions as well as how frequently each of the themes was mentioned, as per the first objective of the study.

**TABLE 1:** Focus group themes (*n* = 42).

Theme	*N*	Theme	*N*	Theme	*N*
Medical history/conditions	14	Hearing	2	Confusion	1
Floor surfaces	10	Inactivity	2	Crutches with worn rubbers	1
Balance/instability	8	Mental health status	2	Deformities	1
Medication	6	Muscle strength	2	Diet	1
Dizziness and vertigo	5	Orientation	2	Gender	1
Vision	4	Orthopaedic problems	2	General personality	1
Alcohol	3	Small dogs	2	Getting up quickly	1
Fear of falling	3	Accessibility of home	1	Post-operative	1
Footwear	3	Age	1	Range of motion of lower limbs	1
Gait	3	Blood pressure	1	Small children	1
Pain	3	Bone density	1	Standing without support	1
Environment	2	Calcification in the eardrum	1	Things lying on the floor	1
Fall history	2	Climbing on a ladder	1	Too much physical support	1
Foot conditions	2	Clothes	1	Walking	1

A total of 42 themes emerged from the data, and the most prominent themes were identified as ‘medical history/conditions’ (*n* = 14), followed by ‘floor surfaces’ (*n* = 10) and ‘balance/instability’ (*n* = 8). One theme, ‘medication’, was mentioned six times and ‘dizziness and vertigo’ five times, followed by ‘vision’ four times. Five fall risk factors were mentioned three times each, 10 factors were mentioned twice and the remaining factors (*n* = 21) were only mentioned once during the discussions. Of the identified themes, four themes could not be linked to the ICF as they were classified as Personal Factors, namely ‘age’, ‘fall history’, ‘gender’ and ‘medical history/conditions’. The remaining 38 themes could be linked to the ICF, resulting in a total of 142 ICF codes, as depicted in Table 2, to satisfy the second objective of the study.

**TABLE 2:** Focus group themes linked to the International Classification of Functioning, Disability and Health.

Body function	ICF code	*N*	Body structure	ICF code	*N*	Activities and participation	Code	*N*	Environmental factors	ICF code	*N*
Seeing	b210	10	Additional musculoskeletal structures related to movement	s770	13	Watching	d110	10	Design, construction; building products and technology of buildings for private use	e155	13
Proprioception function	b260	8	Structure of inner ear	s260	6	Maintaining a body position	d415	5	Products or substances for personal consumption	e110	9
Sensations associated with hearing and vestibular function	b420	7	Structures related to movement, other specified	s798	3	Moving around in different locations	d460	4	Products and technology for personal use in daily living	e115	5
Vestibular functions	b235	6	Structure of the trunk	s760	2	Changing and maintaining body position; other specified and unspecified	d429	2	Domesticated animals	e350	2
Gait pattern	b770	4	Structure of lower extremity	s750	1	Changing basic body position	d410	1	Extended family	e315	1
Emotional functions	b152	4	Structure of external ear	S240	1	Hand and arm use	d445	1	Natural environment and human-made changes to environment; other specified	e298	1
Control of voluntary movement	b760	3	-	-	-	-	-	-	Natural events	e230	1
Sensations of pain	b280	3	-	-	-	-	-	-	-	-	-
Activity level	b125	2	-	-	-	-	-	-	-	-	-
Global psychosocial functions	b122	2	-	-	-	-	-	-	-	-	-
Involuntary movement reaction	b755	2	-	-	-	-	-	-	-	-	-
Muscle power	b730	2	-	-	-	-	-	-	-	-	-
Orientation functions	b114	2	-	-	-	-	-	-	-	-	-
Consciousness function	b110	1	-	-	-	-	-	-	-	-	-
Mobility of joints	b710	1	-	-	-	-	-	-	-	-	-
Perceptual functions	b156	1	-	-	-	-	-	-	-	-	-
Stability of joints	b715	1	-	-	-	-	-	-	-	-	-
Temperament and personality	b126	1	-	-	-	-	-	-	-	-	-
Weight management	b530	1	-	-	-	-	-	-	-	-	-

**Total** **Percentage**	**-** **-**	**61** **43%**	**-** **-**	**-** **-**	**26** **18%**	**-** **-**	**-** **-**	**23 16%**	**-** **-**	**-** **-**	**32** **23%**

ICF, International Classification of Functioning, Disability and Health.

As depicted in Table 2, of the 142 ICF codes identified from the 38 themes mentioned in the discussions, 43% (*n* = 61) were in the Body Function category, 23% (*n* = 32) in the Environmental Factors category, 18% (*n* = 26) in the Body Structure category and 16% (*n* = 23) in the Activities and Participation category.

Differences were calculated between all four categories, and statistically significant differences were found for the comparison between Body Function (*n* = 61) vs Body Structure (*n* = 26) – *p* < 0.0001; Body Function (*n* = 61) vs Activities and Participation (*n* = 23) – *p* < 0.0001; and Body Function (*n* = 61) vs Environmental Factors (*n* = 32) – *p* = 0.0003. No statistically significant differences were reported for any of the other comparisons (*p* > 0.05).

## Discussion

As expected, the results of this study revealed that the main focus of HCPs was on Body Function. The way in which the body functions is important to HCPs, as there is no better indication of successful assessment and intervention outcomes than improved functioning. Difficulties in functioning urge patients to seek advice from HCPs so as to improve their health and increase their own functioning (Bickenbach et al. 2012). When considering the ICF, a person’s functioning (on the level of the body) is important for HCPs, as it describes the outcome of four main health strategies, namely prevention, cure, rehabilitation and support. The ICF also offers a common terminology for the improvement of clinical and patient-orientated assessment instruments (Bickenbach et al. 2012; World Health Organization 2012). A comparison between the perspectives of HCPs and a recent systematic review of FRATs (De Clercq et al. 2020) indicated that the majority of perspectives of both the FRATS and the HCPs focussed on Body Function. It also showed that the knowledge of HCPs was in line with contemporary knowledge in the field.

Functioning is furthermore related to the environment, as it essentially captures the functioning of the body in ‘real-life contexts’ and reflects how the body and the environment interact with one another to either increase or decrease older adults’ ability to function. It was not at all surprising that approximately a quarter of the factors mentioned by the HCPs could be categorised under Environmental Factors. Almost all the activities of daily life are complex and require complex and dynamic interaction with the environment (Young & Williams 2015) (e.g. walking along an uneven pavement or stepping over obstacles on the floor). The physical environment poses the most significant environmental risk for older adults, and often home hazards are the most important to consider in understanding and preventing falls, especially for persons who fall repeatedly (Letts et al. 2010). A person’s interaction with the environment is therefore important, as the type of interaction could increase fall risk.

When considering the number of codes in each ICF category, about a third of the second-level codes are in the Activities and Participation category. This category entails three concepts: one is the task being executed (Activities) (World Health Organization 2002) and the others are two Participation concepts, namely attending (physical presence) and involvement in activities (the type of activities the older adult is participating in, whilst being physically present) (Adair et al. 2018; Imms et al. 2017). In the current study, however, the HCPs had a minimal focus on this category and they only discussed Activity-related factors. They did not include any Participation factors, such as domestic life activities, relationship activities and community or social life activities, in this category. One possible reason for this could be that Activities, the execution of a task, is more closely related to Body Functions, and as such, more in line with the role of HCPs in the clinical identification of fall risk factors. Participation codes, on the other hand, are more in line with intervention strategies, which were not discussed by the focus groups.

A comparison between the clinical perspectives of HCPs on fall risk factors in older adults and the systematic review of FRATs (De Clercq et al. 2020) revealed that the HCPs mentioned two relevant factors that were not captured in existing FRATs, namely ‘muscle-power functions’ and ‘mobility-of-joint functions’. Both of these ICF codes are important to consider for fall risk in older adults, as they relate directly to the ability to execute mobility activities. Almost 25% of older adults have mobility limitations, and both muscle-power functions and (to a degree) mobility functions are modifiable impairment limitations on the mobility of older adults (Bean et al. 2007). Studies have shown a link between lack of mobility and flexibility, and poor walking ability and balance in older adults (Martínez-López et al. 2014). Healthcare practitioners were clearly aware of the importance of these two aspects and included them in the discussions, thus revealing the importance of these clinical perspectives in the discussion of fall risk in older adults.

Healthcare practitioners have a crucial role to play in identifying fall risk factors in older adults and also in assisting older adults to understand the importance of reducing their own risk, not only in terms of their medical conditions but also with regard to their environment and how they engage and participate in activities. Early identification of fall risk factors, combined with appropriate referrals to other HCPs when needed, could reduce older adults’ fall rate by up to 24% (Howcroft et al. 2013; Phelan et al. 2015). Our findings revealed that HCPs’ knowledge is in line with current literature and they were well aware of the importance of including aspects not only related to Body Function, even though the latter was their main focus. By gathering the perspectives of HCPs on the topic at hand, we were able to add the necessary clinical evidence to support the development (in future research) of a holistic instrument to identify fall risk in older adults. Such an instrument could guide intervention strategies for this population, as well as be used by HCPs, in different settings, with ease and consistency. It could ultimately assist HCPs in guiding older adults on how to reduce their own risk of falling.

## Limitations of the study

All data was collected from HCPs in urban Gauteng, although they represented both public and private facilities. The clinical knowledge, skills and experience of these HCPs may, however, differ from those of their peers in smaller towns or rural areas. Furthermore, this research only focussed on the perspectives of one stakeholder group, namely HCPs. Only one general question and a follow-up question were asked to elicit information about the participants’ perspectives. Further studies could consider asking more specific questions regarding each ICF category to elicit more detailed ICF-focussed as well as intervention-related responses.

## Implications and conclusion

Whilst this article provided insight into the perspectives of HCPs on factors contributing to fall risk in older adults, it also demonstrated that HCPs are not only aware of current literature on the topic but also have knowledge of factors not specifically related to the body (i.e. the environment and the older adults’ ability to perform physical activities).

By comparing the perspectives of HCPs on fall risk in older adults to current literature as well as to the perspectives of the older adults themselves, future research could pursue a twofold aim: it could provide a holistic picture of fall risk factors in older adults, and it could use this information to further develop ICF tools to guide the comprehensive management of fall risk in this population. Future research could also identify factors that are focussed on intervention in fall risk management in older adults and determine if the perspectives of HCPs are translated into clinical practice.
